# Enhancement of In Vitro Bioaccessibility of *Alpinia officinarum* Hance Extract With Dual‐Coated Liposomes

**DOI:** 10.1002/fsn3.70438

**Published:** 2025-07-01

**Authors:** İlkyaz Patır, Büşra Karkar, Saliha Şahin

**Affiliations:** ^1^ Department of Chemistry, Faculty of Science and Arts Bursa Uludag University Bursa Türkiye

**Keywords:** *Alpinia officinarum* Hance, bioaccessibility, coatings, flavonoid, liposomes, polymer

## Abstract

Bioaccessibility is a significant problem hindering dietary polyphenols' beneficial effect on human health. Plants, such as spices, have a high polyphenol content, but the human body can only benefit from the part absorbed from the intestine. *Alpinia officinarum* Hance is also a spice with high polyphenol content, but its phenolic compounds have low bioaccessibility with low solubility, stability, and poor intestinal absorption. Although various carrier systems have been developed to overcome this situation, the performance of chitosan–sodium alginate coated liposomes was evaluated for the first time in this study. It was demonstrated that in contrast to various carrier systems developed, double‐coated liposomes preserve the active ingredients and increase their bioaccessibility in environments with different pH, enzyme, and salt conditions. For the enhancement of the bioaccessibility of its phenolic compounds, *Alpinia officinarum* Hance extract was encapsulated in dual‐coated liposomes. For this purpose, chromatographic and spectroscopic analysis of *Alpinia officinarum* Hance extract was performed. The dual‐coated liposomes were produced and characterized, and the in vitro release profile and bioaccessibility were investigated. The significant differences between the results were statistically analyzed. The liposomes were produced with 93.19% ± 0.02% encapsulation efficiency and a size of 155 ± 22.61 nm, zeta potential of −40.97 ± 13.41 mV. The bioaccessibility of galangin in *Alpinia officinarum* Hance extract was 23.87% ± 0.24%, and in dual‐coated liposomes was 73.65% ± 1.70%. By virtue of the developed system, liposomes loaded with *Alpinia officinarum* Hance extract resisted gastrointestinal conditions and increased its bioaccessibility approximately threefold by slowing the release of the extract. The results are statistically significant.

## Graphical abstract

The liposomes were produced with 93.19% ± 0.02% encapsulation efficiency and a size of 155 ± 22.61 nm, zeta potential of −40.97 ± 13.41 mV. The bioaccessibility of galangin in *Alpinia officinarum* Hance extract was 23.87% ± 0.24%, and in dual‐coated liposomes was 73.65% ± 1.70%. By virtue of the developed system, liposomes loaded with *Alpinia officinarum* Hance extract resisted gastrointestinal tract conditions and increased its bioaccessibility approximately three fold by slowing the release of the extract.

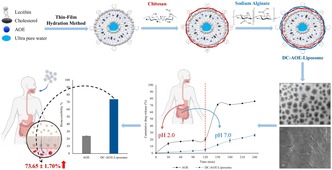

## 1 Introduction

Flavonoids are one of the largest and most diverse subclasses of polyphenols, which are secondary metabolites of plants. Fruits, vegetables, spices, wine, teas, and chocolate are natural sources of flavonoids and have a high and varied content (Carpena et al. 2021). In recent years, many studies on the relationship between flavonoids and health have shown a positive relationship between preventing chronic diseases and consuming flavonoids in the daily diet (Kapolou et al. 2021). For example, the rhizomes of *Alpinia officinarum* Hance, used as a spice (Wang et al. 2022), contain high amounts of flavonoids with high antioxidant activity such as galangin, isorhamnetin, kaempferid, quercetin, chrysin, and kaempferol (He et al. 2025). *Alpinia officinarum* Hance has been reported to have various medicinal activities such as antioxidant, antibacterial, anti‐inflammatory, anti‐cancer, anti‐genotoxic, anti‐fungal and anti‐tumor due to its high bioactive compound content (Awad et al. 2020). Galangin is the main bioactive constituent of rhizomes (Lu et al. 2021), but galangin has a very low bioavailability due to its low water solubility (Abbas et al. 2023; Prasad and Kumari 2022), being easily affected by temperature, pH and light (Lu et al. 2021), in addition to its insufficient oxidative stability and poor intestinal absorption, so its use is limited (Hajipour et al. 2021; Jeong et al. 2016; Li, Peng, et al. 2018; Sabry et al. 2021). The high bioaccessibility of the fraction of a component released from the matrix that can be absorbed in the intestine level of flavonoids in foods is a critical factor for maximizing the benefit, therefore, the bioavailability of flavonoids (Thakur et al. 2020). Lan et al. (2023) reported that most of flavonoids undergo enzymatic degradation in the large intestine, and only 5%–10% are absorbed. They also declare that the low bioavailability led by this low intestinal absorption is the main reason limiting the clinical applications of flavonoids.

Liposomes are frequently used as carriers for flavonoids because they are composed of a phospholipid bilayer mimicking the cell membrane, can encapsulate both hydrophobic and hydrophilic components, and have high mobility due to their small size. In recent studies, nanoliposomes have been frequently used as a flavonoid carrier system (Rambaran 2020). The common opinion reported in these studies is that liposomes increase the bioaccessibility of flavonoids and, indirectly, their bioactivity. For example, Yao et al. (2019) developed galangin‐loaded PEG‐modified liposomes and reported that galangin loaded into liposomes had better solubility and improved anti‐tumor activity and pharmacokinetics compared to free galangin. Similarly, Zhu et al. (2018) developed galangin‐loaded liposomes and reported that the solubility and oral bioavailability of galangin in liposomes were improved and it had a higher hepatoprotective effect compared to free galangin.

However, liposomes may be unstable as a result of external stress factors such as light, temperature change, acidic, and basic environment, and as a result, liposomes may agglomerate, and an increase in particle size may occur, which may cause leakage or degradation of encapsulated compounds. In addition, liposomes can be adversely affected by acids and enzymes in the gastrointestinal medium in the human body (Cui et al. 2021). The liposomal membrane can be destabilized by pH, bile salts and enzymes in the gastrointestinal medium. The ester bonds of phospholipids, which are the basic structural components of liposomes, are hydrolyzed by the acidic pH of the stomach, and lipases, and bile salts act as surfactants, leading to the dissolution of the liposome membrane (Sebaaly et al. 2021). Liposomes provide the possibility and space for surface functionalization to overcome these problems. For this purpose, modifications performed on the surfaces of liposomes using polymers are widely used approaches. Liposomes enable the adsorption of polymers on their outer surfaces through electrostatic, dipole‐charge, hydrogen bonding, van der Waals interactions. Layer‐by‐layer (LbL) deposition of oppositely charged polymers such as chitosan and sodium alginate are the most widely used method (Cao et al. 2022). Alginate is a natural polysaccharide and an anionic biopolymer (Ahmad et al. 2021). Like alginate, chitosan is a natural polysaccharide and a cationic biopolymer due to the amino groups in its structure (Harugade et al. 2023). Chitosan and sodium alginate create a polyelectrolyte complex (Jang et al. 2024) and this stable complex are used for controlled drug release (Harugade et al. 2023).

In our previous study, optimum conditions were determined for developing galangin‐loaded liposomes with maximum encapsulation efficiency, and optimum liposomes were modified with chitosan–gum arabic. The liposomes obtained are neutral liposomes with a zeta potential value of −6.0 ± 4.0 mV. Although neutral liposomes can change their charge according to different pH values, they must have a charge more negative than −30 mV or more positive than +30 mV to ensure the physical stability of the liposomes. The main purpose of this study was to obtain liposomes with a high negative zeta potential. The zeta potential of sodium alginate is more negative than that of gum arabic; therefore, chitosan will interact more electrostatically with sodium alginate than with gum arabic. Therefore, sodium alginate–chitosan coated liposomes will show lower galangin release than the arabic‐chitosan coated liposomes developed in our previous study. This will increase the bioaccessibility of *Alpinia officinarum* Hance extract by providing extra stability in the gastrointestinal medium. In this study, different from our previous study, we focused on chromatographic and spectroscopic analysis of *Alpinia officinarum* Hance extract (AOE) and the bioaccessibility of galangin. This study aimed to increase the bioaccessibility of AOE by encapsulation in dual‐coated liposomes. For this purpose, AOE‐loaded liposomes were prepared using a thin‐film hydration technique and coated with chitosan and alginate using a LbL deposition technique. The obtained AOE‐loaded dual‐coated liposomes (DC‐AOE‐liposome) were characterized, and in vitro gastrointestinal digestion behavior, in vitro release behavior, and bioaccessibility were determined. The main phenolic component of *Alpinia officinarum* Hance extract is the flavonoid galangin. Therefore, the analysis is based on the measurement of galangin.

## 2 Materials and Methods

### 2.1 Material

Acetonitrile in HPLC grade, chitosan (Medium molecular weight), alginic acid sodium salt, and cholesterol were purchased from Sigma‐Aldrich (St. Louis, MO, USA). Formic acid, acetic acid, and ethanol were purchased from Merck (Darmstadt, Germany). *Alpinia officinarum* Hance root powder was purchased from a local producer in Türkiye.

### 2.2 Extraction of *Alpinia officinarum* Hance

For the phenolic compounds extraction from *Alpinia officinarum* Hance (AOE), 1:5 w/v (g/mL), *Alpinia officinarum* Hance root powder:ethanol was stirred in a magnetic stirrer for 4 h at 25°C. The obtained AOE was filtered through Whatman No. 1 filter paper and stored at +4°C for 1 day until used (Patır 2023; Karkar et al. 2023).

### 2.3 Chromatographic Analysis of *Alpinia officinarum* Hance

The phenolic compounds profile AOE was determined by the HPLC‐DAD analysis. Briefly, AOE diluted 1:10 ratio, and the phenolic compound of AOE was determined by HPLC‐DAD (Agilent 1200 Series, USA). The XBridge C18 (4.6 × 250 mm, 3.5 μm) column (Waters, USA) was used for chromatographic separation of the phenolic compound. The analysis conditions of HPLC: 10 μL of injection volume, 0.5 mL/min of flow rate, 36 min of run time, 280–360 nm of wavenumber range, the gradient mobile phase (1% formic acid in water and acetonitrile). The quantitation of the phenolic compound was calculated by a standard calibration curve. Standard galangin solutions in the concentration range of 1–40 ppm were used to create the calibration graph. The line equation of the calibration graph was *y* = 52.487*x* − 3.4043 and *R* ^2^ = 0.9994. The limit of detection (LOD) was 0.01 mg/L and limit of quantitation (LOQ) was 0.02 mg/L. Standard quercetin solutions in the concentration range of 2–20 ppm were used to create the calibration graph. The line equation of the calibration graph was *y* = 58.096*x* + 4.1535 and *R* ^2^ = 1. The LOD was 0.01 mg/L and LOQ was 0.02 mg/L. Standard kaempferol solutions in the concentration range of 2–20 ppm were used to create the calibration graph. The line equation of the calibration graph was *y* = 55.922*x* − 0.3611 and *R* ^2^ = 0.9999. The LOD was 0.01 mg/L and LOQ was 0.03 mg/L. Standard isorhamnetin solutions in the concentration range of 2–20 ppm were used to create the calibration graph. The line equation of the calibration graph was *y* = 69.985*x* − 26.606 and *R* ^2^ = 0.9999. The LOD was 0.01 mg/L and LOQ was 0.02 mg/L. Standard chrysin solutions in the concentration range of 2–20 ppm were used to create the calibration graph. The line equation of the calibration graph was *y* = 98.296*x* − 3.6417 and *R* ^2^ = 0.9993. The LOD was 0.01 mg/L and LOQ was 0.06 mg/L (Karkar and Şahin 2022).

### 2.4 Spectroscopic Analysis of AOE

The spectroscopic analyses of AOE were performed using three methods. The total phenolic content of AOE was analyzed using the Folin–Ciocalteu method, total antioxidant capacity using the ABTS method, and total flavonoid content using the Aluminum chloride method.

In the Folin–Ciocalteu method, 50:1 Na_2_CO_3_ (1%) in 0.1 M NaOH and 0.5% CuSO_4_ in 1% NaKC_4_H_4_O_6_ were mixed. The mixture was added to a test tube containing 0.1 mL of AOE and 1.9 mL of ultrapure water, and 0.25 mL of Folin–Ciocalteu reagent. After 30 min incubation, the absorbance of the samples was measured at 750 nm by UV–Vis spectrophotometer (Varian, Cary 50 Conc). As a result of the analysis, the total phenolic content was expressed as mg gallic acid equivalent (GAE)/g sample. Gallic acid solutions in the concentration range of 1–60 ppm were used to create the calibration graph. The line equation of the calibration graph was *y* = 0.0314*x* − 0.0015 and *R* ^2^ = 0.9978 (Karkar and Şahin 2022).

In the ABTS method, 0.1 mL of AOE and 3.9 mL of ethanol were mixed, and 1 mL of ABTS^•^ radical solution was added. After 6 min of incubation, sample absorbance was measured at 734 nm in a UV–Vis spectrophotometer. As a result of the analysis, the total antioxidant capacity was expressed as mg trolox equivalent (TE)/g sample. Trolox solutions in the concentration range of 0.3–10 ppm were used to create the calibration graph. The line equation of the calibration graph was *y* = 8.8122*x* − 2.2937 and *R* ^2^ = 0.9981 (Karkar and Şahin 2022).

In the Aluminum chloride method, 0.5 mL of AOE, 1.5 mL of ethanol (95%), 0.1 mL of AlCl_3_·6H_2_O (10%), and 0.1 mL of NaC_2_H_3_O_2_·3H_2_O (1 M) were mixed with ultrapure water to a total volume of 5 mL. The samples were incubated at room temperature for 40 min and then their absorbance was measured at 415 nm in a UV–Vis spectrophotometer. As a result of the analysis, the total flavonoid amount was expressed as mg quercetin equivalent (QE)/g sample. Quercetin solutions in the concentration range of 0.5–10 ppm were used to create the calibration graph. The line equation of the calibration graph was *y* = 0.1053*x* − 0.0061 and *R* ^2^ = 0.9996 (Formagio et al. 2014).

### 2.5 Preparation of DC‐AOE‐Liposome

The AOE‐loaded liposomes were prepared using the thin film hydration method. Our previous study, the liposome production parameters were optimized using central composite design‐response surface methodology. The optimization parameters soybean lecithin/solvent ratio (1–9 mg/mL), cholesterol/solvent ratio (0.2–1.8 mg/mL), time (15–55 min), and galangin/solvent ratio (0.1–0.5 mg/mL) was investigated with 30 experiments. In this study, the liposome composition was selected as soybean lecithin:cholesterol:galangin, 1:1:0.15, which had the highest encapsulation efficiency (93.19%) in the experimental design table in our previous study (Karkar et al. 2023). Briefly, soybean lecithin, cholesterol, and AOE were dissolved in 25 mL of ethanol in a 1:1:0.15 ratio and sonicated in an ultrasonic bath at 40°C for 35 min. After sonication, ethanol was evaporated at 55°C until a thin film was obtained. The thin film was then hydrated with 10 mL of ultrapure water and filtered through a 0.45 μm PVDF filter (Zhu et al. 2018).

The AOE‐loaded liposomes were coated by the LbL deposition method. Briefly, 10 mL of liposomes were added dropwise into 10 mL of chitosan solution (0.625%, pH 5.5) by syringe and mixed at 200 rpm for 1 h. After 1 h, the pH was adjusted to pH 5.5 and centrifuged for 30 min. For the coating of chitosan‐coated liposomes with alginate, 10 mL of chitosan‐coated optimum liposome solution was added dropwise into 10 mL of alginate solution (2%, pH 5.5) and mixed at 200 rpm for 1 h. After 1 h, the pH was adjusted to pH 5.5 and centrifuged for 30 min. The obtained DC‐AOE‐Liposome were stored in the refrigerator at +4°C until use (Liu et al. 2013).

### 2.6 Encapsulation Efficiency of DC‐AOE‐Liposome

The encapsulation efficiency (EE) % of DC‐AOE‐liposomes was determined by the HPLC‐DAD analysis. Briefly, 10 mL of liposome was centrifuged at 4427 *g* RCF for at 4 min (ultracentrifuge tubes, 10 kDa). The supernatant was diluted 1:10 ratio and analyzed by HPLC‐DAD. The quantitation of galangin was calculated by standard galangin calibration curve. EE % was calculated using the following equation:

$$\mathrm{EE}\%=\frac{\text{Amount of galangin in}\;\mathrm{DC}‐\mathrm{AOE}‐\text{liposome}}{\text{Initial amount of galangin}}\times 100$$

### 2.7 Characterization of DC‐AOE‐Liposome

The hydrodynamic diameter, polydispersity index, and zeta potential of DC‐AOE‐liposome were determined using the Malvern Zetasizer Nano‐ZS based on dynamic light scattering (DLS). The samples were homogenized in an ultrasonic bath for 30 min and then diluted at a 1:10 ratio with ultrapure water. The diluted samples were sonicated for 10 min, and measurements were performed at 25°C.

The surface morphology and size of DC‐AOE‐liposome were determined by field emission scanning electron microscopy (FE‐SEM). The liposome samples were lyophilized before analysis, and imaging was performed at 3.00 kV resolution and 20.00 KX magnification. The surface morphology of liquid DC‐AOE‐liposome was determined by transmission electron microscopy (TEM). The TEM analysis was performed at 120.00 kV resolution and 5.00 KX magnification.

### 2.8 In vitro Release Study

The dialysis method was used to examine the release behavior of galangin from DC‐AOE‐liposomes under in vitro simulated physiological conditions. Briefly, 10 mL of DC‐AOE‐liposome and AOE containing an equivalent amount of galangin loaded into DC‐AOE‐liposome as control were placed in dialysis bags. The dialysis bags were placed in flasks containing phosphate‐buffered saline (PBS, pH 7.4) solution. The flasks were placed on a temperature‐programmed shaker set at 37°C and 100 rpm shaking speed, and 3 mL of sample was taken from the flasks every 30 min for 450 min, and 3 mL of PBS was added to the flasks. Then, the samples were taken into ultracentrifuge tubes (10 kDa) and centrifuged at 4427 *g* RCF for 3 min, and the absorbance of the samples was read at 360 nm in a UV–Vis spectrophotometer. Experiments were carried out with three replicates for each time period (Jayan et al. 2019). Significant differences between the cumulative drug release during in vitro digestion of AOE and DC‐AOE‐liposome were statistically analyzed with ANOVA Fit General Linear Model at *p* < 0.01 confidence level using MINITAB 17.0 (Minitab Inc., State College, PA, USA) statistical program.

### 2.9 In vitro Gastrointestinal Digestion Study

The release behavior of DC‐AOE‐liposome in the in vitro simulated gastrointestinal environment was studied by modifying the methods of Maria Leena et al. (2020), 10 mL of DC‐AOE‐liposome and 10 mL of AOE containing an equivalent amount of galangin loaded into DC‐AOE‐liposome as control were placed in flasks and 10 mL of simulated gastric fluid (SGF) (2000 U/mL of pepsin) (pH 2.0) was added. They were mixed in a temperature‐programmed shaker at 100 rpm for 2 h at 37°C. Every 30 min, 1 mL of sample was taken into the test tubes, 1 mL of SGF was added to the flasks, and the reaction was stopped by placing the test tubes in a cold‐water bath. At the end of simulated gastric digestion, pH was adjusted to 7.0, and 20 mL of simulated intestinal fluid (SIF) (100 U/mL of pancreatin and 10 mM bile salt) (pH 7.0) was added to the flasks for simulated intestinal digestion. The same steps mentioned above were performed for simulated intestinal digestion under the same conditions using SIF instead of SGF in temperature‐programmed shaker at 100 rpm for 2 h at 37°C. At the end of the gastrointestinal digestion, Then, the samples were taken into ultracentrifuge tubes (10 kDa) and centrifuged at 4427 *g* RCF for 3 min, and the absorbance of the supernatant was measured at 360 nm in a UV–Vis spectrophotometer. Experiments were carried out with three replicates for each time period. Significant differences between the cumulative drug release during in vitro gastrointestinal digestion of AOE and DC‐AOE‐liposome were statistically analyzed with ANOVA Fit General Linear Model at *p* < 0.01 confidence level using MINITAB 17.0 (Minitab Inc., State College, PA, USA) statistical program.

### 2.10 Bioaccessibility Study

Bioaccessibility of galangin in DC‐AOE‐liposome and AOE was performed as described in Section 2.9. Briefly, 10 mL of SGF (pH 2.0) was added to 10 mL of DC‐AOE‐liposome and 10 mL of AOE and shaken in a temperature‐programmed shaker at 100 rpm for 37°C for 2 h. At the end of the 2 h gastric digestion, a 1 mL sample was taken, and the reaction was stopped by placing the test tubes in a cold‐water bath. At the end of simulated gastric digestion, the pH was adjusted to 7.0, and 20 mL of SIF (pH 7.0) was added and shaken in a temperature‐programmed shaker at 100 rpm for 37°C for 2 h. At the end of the 2 h intestinal digestion, a 1 mL sample was taken, and the reaction was stopped by placing the test tubes in a cold‐water bath. Then, the samples were taken into ultracentrifuge tubes (10 kDa) and centrifuged at 4427 *g* RCF for 3 min to isolate the micelle phase. Experiments were carried out with three replicates. The following equation was used to calculate the bioaccessibility of galangin (Li, Guo, et al. 2018).

$$\text{Bioaccessibility}\;\left(\%\right)=\frac{\text{Amount of galangin in micelle phase}}{\text{Amount of galangin in liposome}}\times 100$$

Significant differences between the bioaccessibility (%) of AOE and DC‐AOE‐liposome were statistically analyzed with ANOVA fit general linear model at *p* < 0.01 confidence level using MINITAB 17.0 (Minitab Inc., State College, PA, USA) statistical program.

## 3 Result and Discussion

### 3.1 Chromatographic Analysis of *Alpinia officinarum* Hance

**FIGURE 1:**
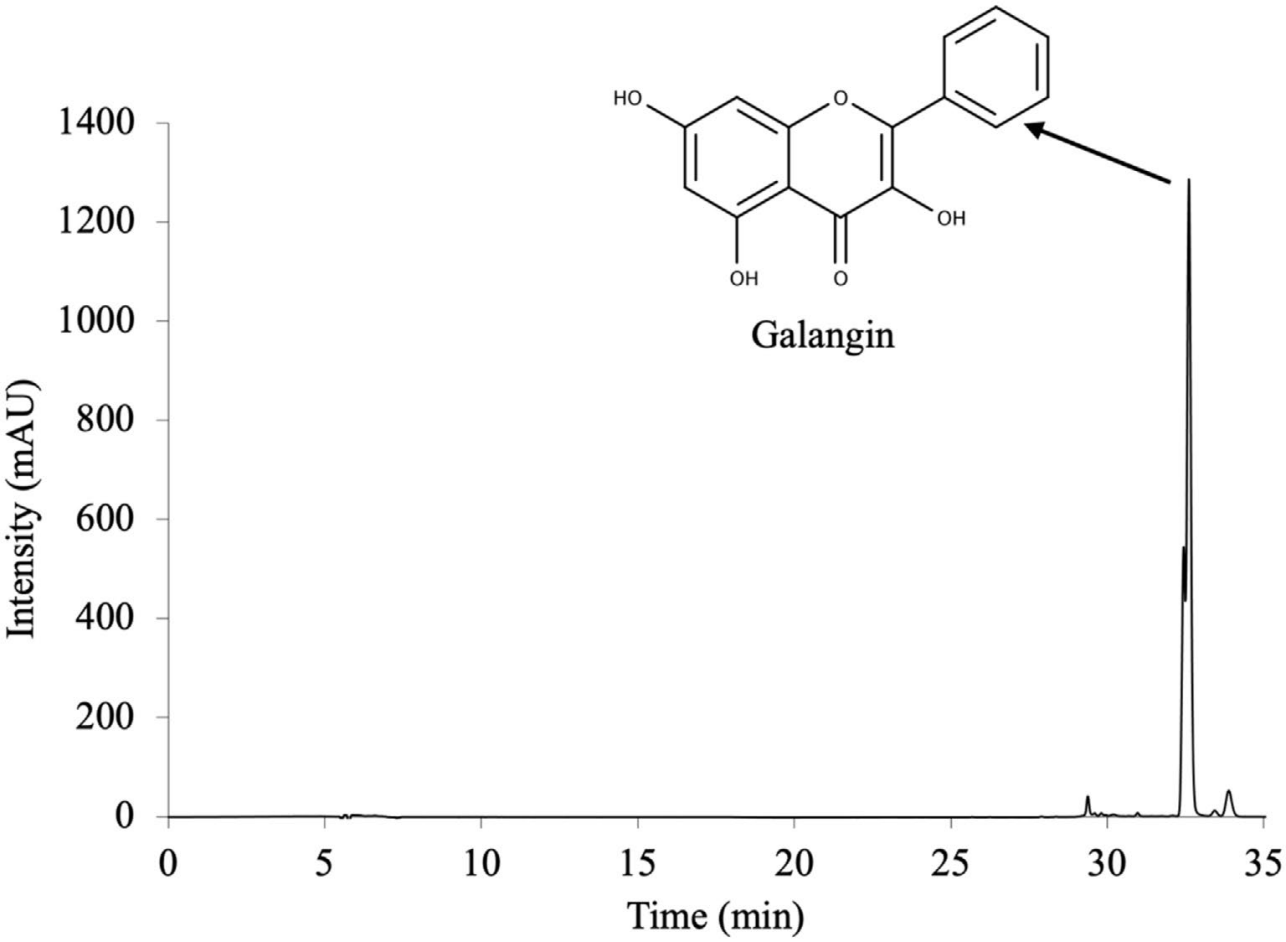
HPLC‐DAD chromatogram of *Alpinia officinarum* Hance.

The chromatogram obtained by HPLC‐DAD analysis for determining phenolic compounds in AOE is given in Figure 1. According to the chromatogram, the component with a retention time of 32.61 min was galangin. The most intense peak detected in the HPLC chromatogram belongs to galangin, and the main component of AOE is galangin. The other peaks in the chromatogram belong to quercetin, kaempferol, isorhamnetin, and chrysin. A study by Jiao et al. (2019), determined the phenolic compound profile of 95% ethanol extract of *Alpinia officinarum* Hance rhizomes collected from 15 different regions by HPLC‐DAD. They reported that the *Alpinia officinarum* Hance extracts contained phenolic compounds such as galangin, galangin‐3‐methyl ether, pinobanksin, and campferide‐4‐methyl ether. When the HPLC chromatogram was analyzed, it was observed that the most substantial peak belonged to galangin. Similarly, Lin et al. (2020), reported that the main component of 80% ethanol extract, ethyl acetate extract (Fang et al. 2018), and 95% *Alpinia officinarum* Hance ethanol extract was galangin (Li, Cheng, et al. 2021).

The amount of galangin in the AOE was calculated from the standard galangin calibration graph and AOE contains 11.57 ± 0.01 mg/g galangin. Also, AOE contains 2.13 ± 0.01 μg/g quercetin, 5.72 ± 0.01 μg/g kaempferol, 3.07 ± 0.01 μg/g isorhamnetin and 43.28 ± 0.01 μg/g chrysin. Tao et al. (2006) reported that the galangin content of methanol extract of *Alpinia officinarum* Hance rhizomes collected from 12 different regions varied between 2.63 and 11.60 mg/g. Jiao et al. (2019) reported that the ethanol extract of *Alpinia officinarum* Hance rhizomes collected from 15 different regions contained galangin between 5.46 and 13.14 mg/g. Pirzadeh et al. (2021) determined that the amount of galangin in the hydroalcoholic (40% ethanol) extract of *Alpinia officinarum* rhizomes was 5.8 mg/g as a result of HPLC analysis. In our previous study, *Alpinia officinarum* Hance was extracted with 80% ethanol and 80% methanol. The 80% ethanol extract contained 11.43 ± 0.07 mg/g galangin, 6.10 ± 0.10 μg/g *p*‐coumaric acid, 61.20 ± 1.50 μg/g quercetin and 219.90 ± 1.50 μg/g kaempferol while the 80% methanol extract contained 10.49 ± 0.11 mg/g galangin, 3.10 ± 0.20 μg/g *p*‐coumaric acid, 60.24 ± 0.20 μg/g quercetin and 196.80 ± 1.80 μg/g kaempferol (Şahin et al. 2024). In general, ethanol, methanol and their combinations with water are preferred as extraction solvents, but the highest galangin content is obtained with ethanol. In addition, galangin content increases with the application of purification, separation and enrichment processes after extraction. For example, Fang et al. (2018) suspended the ethanol extract of *Alpinia officinarum* Hance rhizomes with water and extracted them again with ethyl acetate. Then, they separated 107 mg galangin and 29 mg kaempferide from 270 mg ethyl acetate extract using HSCCC. This corresponds to about 40% of the galangin content of the extract.

### 3.2 Spectroscopic Analysis of AOE

Spectroscopic analysis investigated the total phenolic‐flavonoid content and antioxidant capacity of AOE. The results of the spectroscopic analysis of AOE are given in Table 1.

**TABLE 1:** The spectroscopic analysis result of AOE.

Total phenolic content (mg GAE/g)	Total antioxidant capacity (mg TE/g)	Total flavonoid content (mg QE/g)
113.610 ± 3.285	28.812 ± 0.007	5.759 ± 0.064

Accordingly, the total phenolic content of AOE was determined as 113.610 ± 3.285 mg GAE/g, total antioxidant capacity as 28.812 ± 0.007 mg TE/g, and total flavonoid content as 5.759 ± 0.064 mg QE/g. In a similar approach to our study, Xia et al. (2010) determined the total phenolic and flavonoid contents of 70% ethanolic extract of *Alpinia officinarum* rhizomes. The study reported that the extract contained 359.60 ± 6.10 mg GAE/g total phenolic and 289.90 ± 5.30 mg RE/g flavonoid content. Srividya et al. (2010) prepared a 50% ethanol extract of *Alpinia officinarum* by cold maceration and hot maceration process. Accordingly, the extract prepared by hot maceration had a total phenolic content of 50.10 mg/g and a total flavonoid content of 54.02, while the extract prepared by cold maceration had 41.35 and 36.36 mg/g, respectively. Ravipati et al. (2012) reported that the total phenolic content of *Alpinia officinarum* ethanol extract was 23.35 ± 3.14 mg GAE/g, and the total flavonoid content was 87.39 ± 0.98 mg QE/g. Considering the above examples, although these analysis were carried out using *Alpinia officinarum* Hance rhizomes, different results were obtained like in this study. This means that even though the same plant and plant part are used, the place where the plant is grown, the growing conditions, and the processing steps are different. In addition, the extraction technique and solvent used are the main reasons for the diversity of the plant extract's total phenolic‐flavonoid substance content and antioxidant capacity.

### 3.3 Encapsulation Efficiency of DC‐AOE‐Liposome

Encapsulation efficiency (EE) is one of the most important factors indicating the success of liposomal drug delivery systems. High EE is the target parameter in liposome production for maximum utilization of the active ingredient loaded in liposomes. In this study, the ratio of galangin in the designed liposome formulation was 0.15, corresponding to 3.75 mg. The EE of the liposomes was 93.19% ± 0.02%, indicating that approximately 3.49 mg galangin was loaded into the liposomes. This EE is relatively high in the thin film hydration method because the low EE is seen as a disadvantage of the thin film hydration method. However, contrary to this view, many authors have reported that this is not the case for lipophilic components such as galangin. Zhu et al. (2018) produced galangin‐loaded liposomes with 92.36% EE by the thin film hydration method. They reported that the high EE could be due to the high interaction of galangin with the hydrophobic regions of soy phosphatidylcholine. Landi‐Librandi et al. (2012) encapsulated lipophilic components such as galangin, quercetin, myricetin, and kaempferol into liposomal systems containing soy phosphatidylcholine and cholesterol. The EE of galangin was found in the range of 60%–80% and 80%–95%. Galangin was loaded into liposomes with the highest EE among these components. In another study, Landi‐Librandi et al. (2011) produced liposomal systems using the same components and reached a similar result and reported that liposomes containing galangin had the highest EE and myricetin, the most hydrophilic component, had the lowest EE. As a result of this study, they stated that the reason for the EE was that the interaction of galangin, the most lipophilic component, with the hydrophobic regions of liposomes was higher than the less lipophilic and hydrophilic components. The high EE obtained in this study is compatible with other studies and also shows that high lipophilicity, which is the most significant disadvantage in the effective use of galangin, is an advantage for entrapment into liposomes.

### 3.4 Characterization of DC‐AOE‐Liposome

**FIGURE 2:**
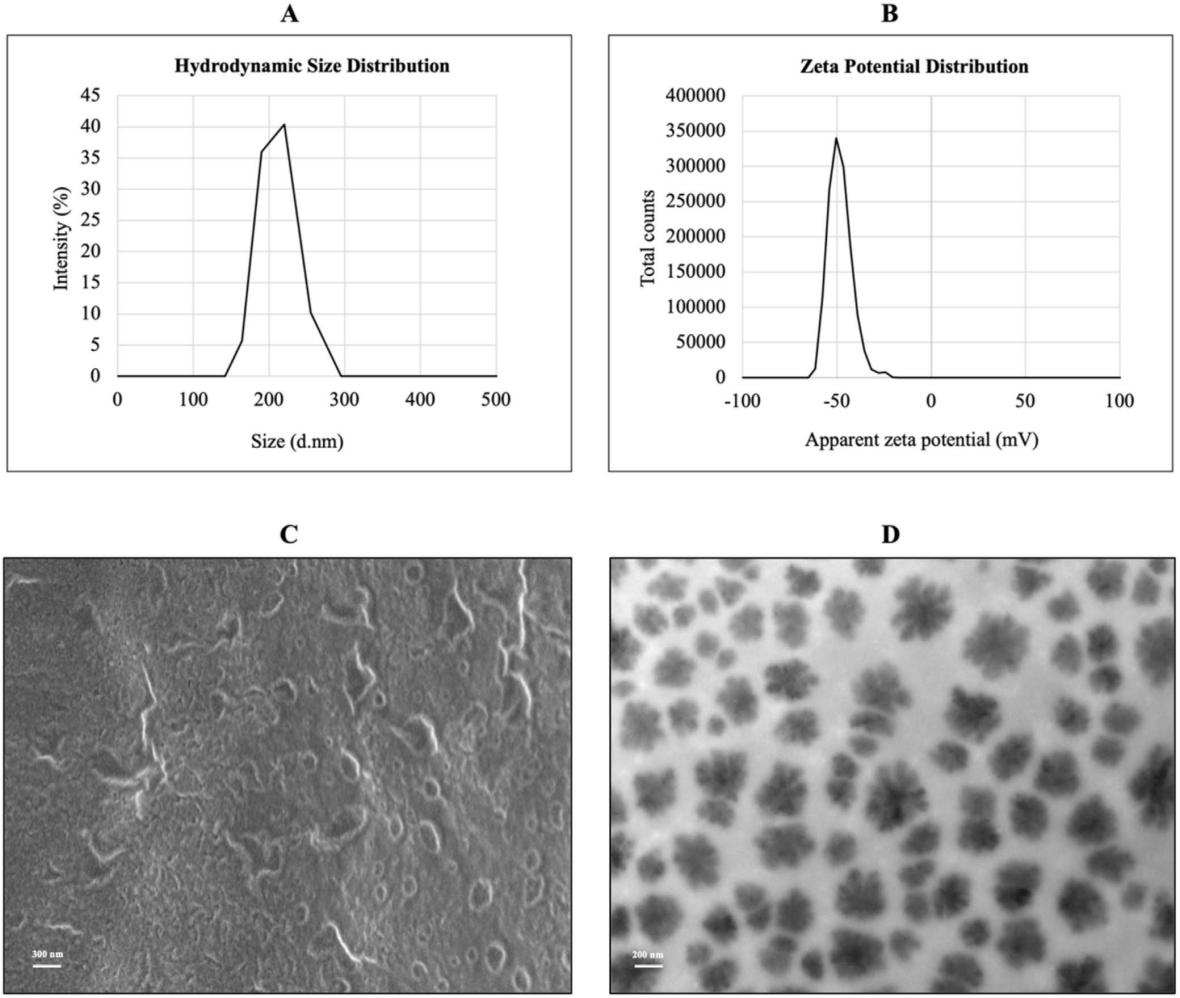
Characterization of DC‐AOE‐liposome. (A) Hydrodynamic size distribution graph of DC‐AOE‐liposome, (B) zeta potential distribution graph of DC‐AOE‐liposome, (C) SEM image of DC‐AOE‐liposome (300 nm, 3.00 kV, 20.00 KX), (D) TEM image of DC‐AOE‐liposome (200 nm, 120.00 kV, 5.00 KX).

The size, hydrodynamic size, zeta potential, polydispersity index, and shape are important parameters in liposome characterization. The characterization of DC‐AOE‐liposome is given in Figure 2 and Table 2.

**TABLE 2:** The characterization of DC‐AOE‐liposome.

Size (nm)	Hydrodynamic diameter (nm)	Zeta potential (mV)	PDI
155.78 ± 22.61	207.37 ± 39.14	−40.97 ± 13.00	0.93

The hydrodynamic size of the DC‐AO‐liposome (Figure 2A) was 207.38 ± 39.14 nm. The hydrodynamic size of DC‐AOE‐liposomes shows a broad distribution between 164.20 and 255 nm. DC‐AOE‐liposomes were developed using a LbL coating technique with polyelectrolyte polymers, and this system is very prone to aggregation due to the swelling of polymers in aqueous media. Madrigal‐Carballo et al. (2010) observed the aggregation phenomenon in chitosan–dextran sulfate‐coated liposomes loaded with ellagic acid. They observed aggregates larger than 3000 nm when coating ellagic acid‐loaded liposomes (300 nm) with chitosan. The aggregate formation was observed when liposomes were coated with a layer of dextran sulfate after chitosan. Accordingly, when two surfaces come into contact, a double layer of electric charge is formed at the interface, and then adhesion develops due to the attractive force arising from electron transfer across the electrical double layer, resulting in the aggregation of particles and the formation of aggregates. This can be seen as the reason for the wide range of hydrodynamic sizes of DC‐AOE‐liposomes.

The zeta potential of the DC‐AOE‐liposome was −40.97 ± 13.41 mV (Figure 2B). This zeta potential value indicates that DC‐AOE‐liposome is an anionic liposome. Liposomes are considered physically and chemically stable if their zeta potential is more negative than −30 mV (Wang et al. 2020). According to this statement, DC‐AOE‐liposome is stable at the obtained zeta potential value. Similar to our result, Sun et al. (2023) determined that the zeta potential values of the chitosan–alginate coated liposomes developed in their study were higher than −30 mV and reported that chitosan–alginate coated liposomes were stable. The polyelectrolytes used for coating liposomes by the LbL method are chitosan, a cationic, and sodium alginate, an anionic polymer. In our previous study, the liposome we developed with soy phosphatidylcholines was negatively charged, but this charge turned positive with chitosan loaded on the surface. The surface charge was neutralized by gum arabic loaded on the chitosan‐loaded surface. Due to its natural structure, sodium alginate has a much stronger negative zeta potential than gum arabic (Barbosa et al. 2019; Li, Zhai, et al. 2021; Rabelo et al. 2019). Therefore, the obtained sodium alginate–chitosan‐coated liposomes have much higher negative zeta potential and structural stability compared to our previous study.

The polydispersity index (size distribution) of the DC‐AOE‐liposome is 0.93. This value indicates that the size distribution of liposomal dispersion was a wide range. Usually, the polydispersity index is expected to be in the range of 0–0.5, indicating the homogeneity of liposome size distribution. This value can have a maximum value of 1, increasing the size distribution's heterogeneity as it approaches 1 (Wen et al. 2014). Liu et al. (2017) and Tu et al. (2023) also reported that PDI values showed that the particle size was not uniform due to modifying the liposome surface with sodium alginate and chitosan with LbL. Gu et al. (2022), reported that the PDI of the alginate chitosan‐coated bamboo leaf flavonoid‐loaded liposomes was 0.36; this value represents the homogeneous dispersion. When SEM and TEM images are also examined, it is observed that the sizes of DC‐AOE‐liposomes are not homogeneously distributed. This confirms the high PDI value obtained for DC‐AOE‐liposomes. A high polydispersity index can cause liposomes to flocculate and aggregate, which negatively affects their stability. Optimizing the amount of polymers used may be a solution to achieve a low PDI (Hiorth et al. 2023).

The FE‐SEM and TEM images of DC‐AOE‐liposome were given in Figure 2C,D. The size of DC‐AOE‐liposomes was determined using FE‐SEM because the hydrodynamic size and TEM analyses in liquid media can introduce dimensional errors due to the swelling of alginate and chitosan. To eliminate these errors, DC‐AOE‐liposomes were lyophilized to solid form prior to FE‐SEM analysis. The size of the DC‐AOE‐liposome was 155.78 ± 22.61 nm (Figure 2C). The fact that liposomes are larger than 100 nm and unilamellar indicates that they are large unilamellar vesicles. Large vesicles usually form when the thin film hydration technique is applied (Andra et al. 2022). Another important parameter in liposome characterization is shape. When FE‐SEM and TEM images were examined, it was seen that the shapes of DC‐AOE‐liposomes were close to spherical. Due to the use of lyophilized DC‐AOE‐liposomes for FE‐SEM images, some distortions occurred in their shapes, but liposomes with a more regular structure were seen in TEM images.

### 3.5 In vitro Release Study

**FIGURE 3:**
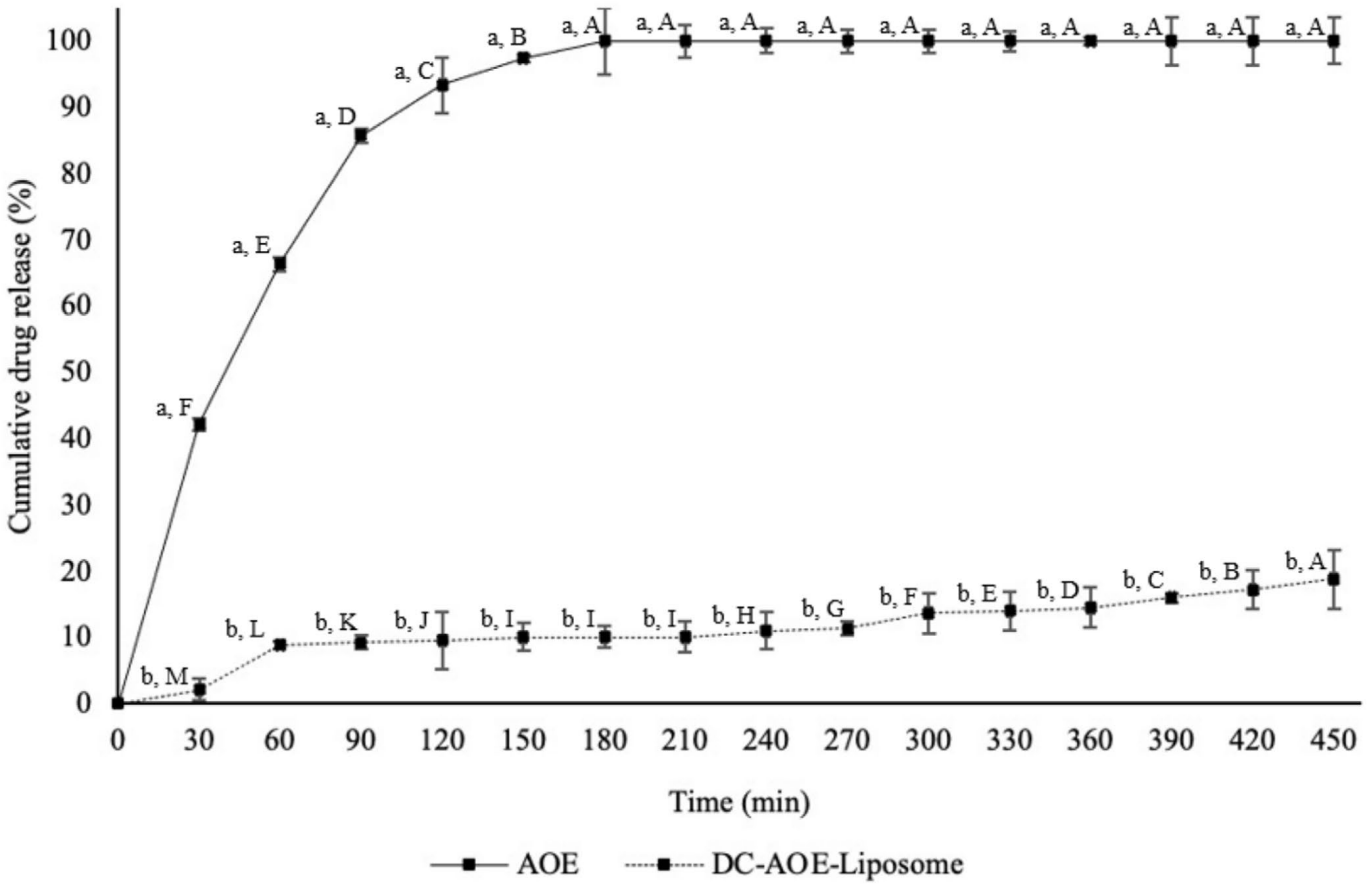
In vitro cumulative release graph of AOE and DC‐AOE‐liposome. (A–M) Uppercase letter indicates significant differences in release time for AOE and DC‐AOE‐liposome. (a, b) Lowercase letter indicates significant differences between AOE and DC‐AOE‐liposome at each release time.

The in vitro release graph of galangin in simulated physiological conditions is given in Figure 3.

Galangin release from AOE reached almost 100% at 150 min, and at the same time, galangin release from DC‐AOE‐liposomes was 8.65% ± 3.70%. At the end of 450 min, which is the end of the release study, galangin release from DC‐AOE‐liposomes was 11.15% ± 1.56%. While 3.49 mg galangin was present in AOE and DC‐AOE‐liposomes at the beginning, all galangin was released from AOE and 0.39 mg galangin was released from DC‐AOE‐liposomes at the end of the study. Surface modification of liposomes with alginate and chitosan slowed down and controlled the release of galangin under simulated physiological conditions. In contrast to simulated gastrointestinal conditions, the release rate is much lower due to the absence of enzymes and salts in the environment, and the polyelectrolyte coating is resistant at pH 7.4. Similar to our study, Gu et al. (2022) developed alginate–chitosan‐coated liposomes loaded with bamboo leaf flavonoids and examined their release under in vitro physiological conditions. At the end of the 24 h study, almost all of the free bamboo leaf flavonoids were released. At the same time, this rate was 55.33% in liposomes, 45.39% in chitosan‐coated liposomes, and 40.32% in alginate–chitosan‐coated liposomes. Based on these results, coating the liposome surface with carbohydrate polymers could slow the release of flavonoids and provide controlled release.

### 3.6 In vitro Gastrointestinal Digestion Study

**FIGURE 4:**
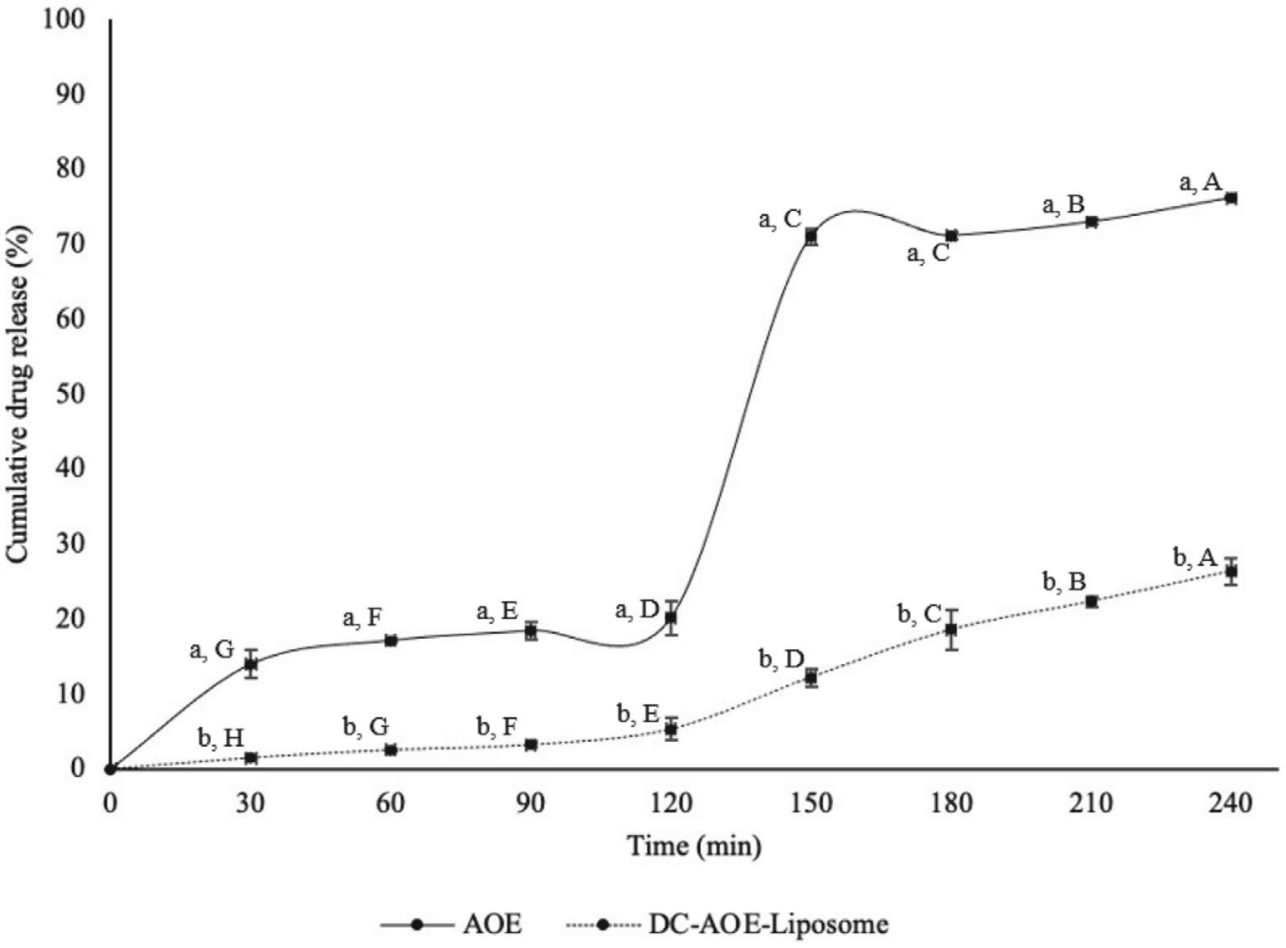
In vitro gastro‐intestinal digestion graph of AOE and DC‐AOE‐liposome. (A–H) Uppercase letter indicates significant differences in release time for AOE and DC‐AOE‐liposome. (a, b) Lowercase letter indicates significant differences between AOE and DC‐AOE‐liposome at each release time.

The stability of liposomes in gastric fluid is essential for the transport of more active substances for intestinal digestion because it is a common phenomenon that the liposomal structure is damaged due to pancreatic enzymes and salts in the composition of the intestinal fluid, and the active substance is released at a high rate. In order for liposomes to be absorbed from the intestine and enter the body circulation and to maximize the bioaccessibility level by maximally transporting active substances to the targeted area, they must resist the gastrointestinal system. The in vitro gastrointestinal digestion graph of AOE and DC‐AOE‐liposome is given in Figure 4. The graph shows that the amount of galangin released from AOE at the end of gastric digestion was 20.13%, while the amount released from DC‐AOE‐liposomes was 5.30%. Accordingly, at the beginning of in vitro gastrointestinal digestion, 3.49 mg galangin was present in AOE and DC‐AOE‐liposomes, and finally, at the end of gastric digestion, the amount of galangin released from AOE was 0.70 mg and from DC‐AOE‐liposomes was 0.18 mg. After gastric digestion, there was a very high increase (approximately 3.5‐fold) in the amount of galangin released from AOE during intestinal digestion (approximately 4‐fold). However, this is not as severe for galangin released from DC‐AOE‐liposomes.

As a result of intestinal digestion, there is a high difference between the amount of galangin released from AOE and DC‐AOE‐liposomes. The galangin release was 76.13% ± 0.24% in AOE and 26.35% ± 1.70% in DC‐AOE‐liposomes, indicating the release of 2.66 mg of galangin from AOE and 0.92 mg of galangin from DC‐AOE‐liposomes at the end of intestinal digestion. These results demonstrate that the LbL technique was successfully applied to liposomes, and the obtained liposome DC‐AOE‐liposome protects galangin by showing resistance against in vitro gastrointestinal digestive conditions. Polymers are favored as liposome coatings due to their enhanced mucoadhesive properties that can increase the adhesion and penetration of drugs across gastrointestinal barriers. Polymers increase the adhesion and penetration of active compounds to gastrointestinal barriers due to their enhanced mucoadhesive properties and are therefore often preferred as coatings in surface modifications of liposomes. When liposomes are coated with charged polymers, they are electrostatically repulsive and stable in colloidal systems. In addition, the polymer coating acts as a barrier that controls the release rate of active compounds (Pasarin et al. 2023). Many studies have reported that liposomes modified by the LbL method successfully protect active ingredients from in vitro gastrointestinal digestive conditions. As an example of this studies Song et al. (2023) reported that liposomes were more affected by low acidic pH than modified liposomes during gastric digestion, and the polymer layer on the surface of modified liposomes protected the liposomes and slowed the release of active substances. In intestinal digestion, they reported that high release rates were achieved because pancreatin accelerated the hydrolysis of phospholipids and bile salts accelerated the breakdown of the liposome membrane, but due to the hardness of the polymer layer on the surface of the modified liposomes, the permeability of enzymes and salts was reduced, and a lower rate of release occurred.

### 3.7 Bioaccessibility Study

**FIGURE 5:**
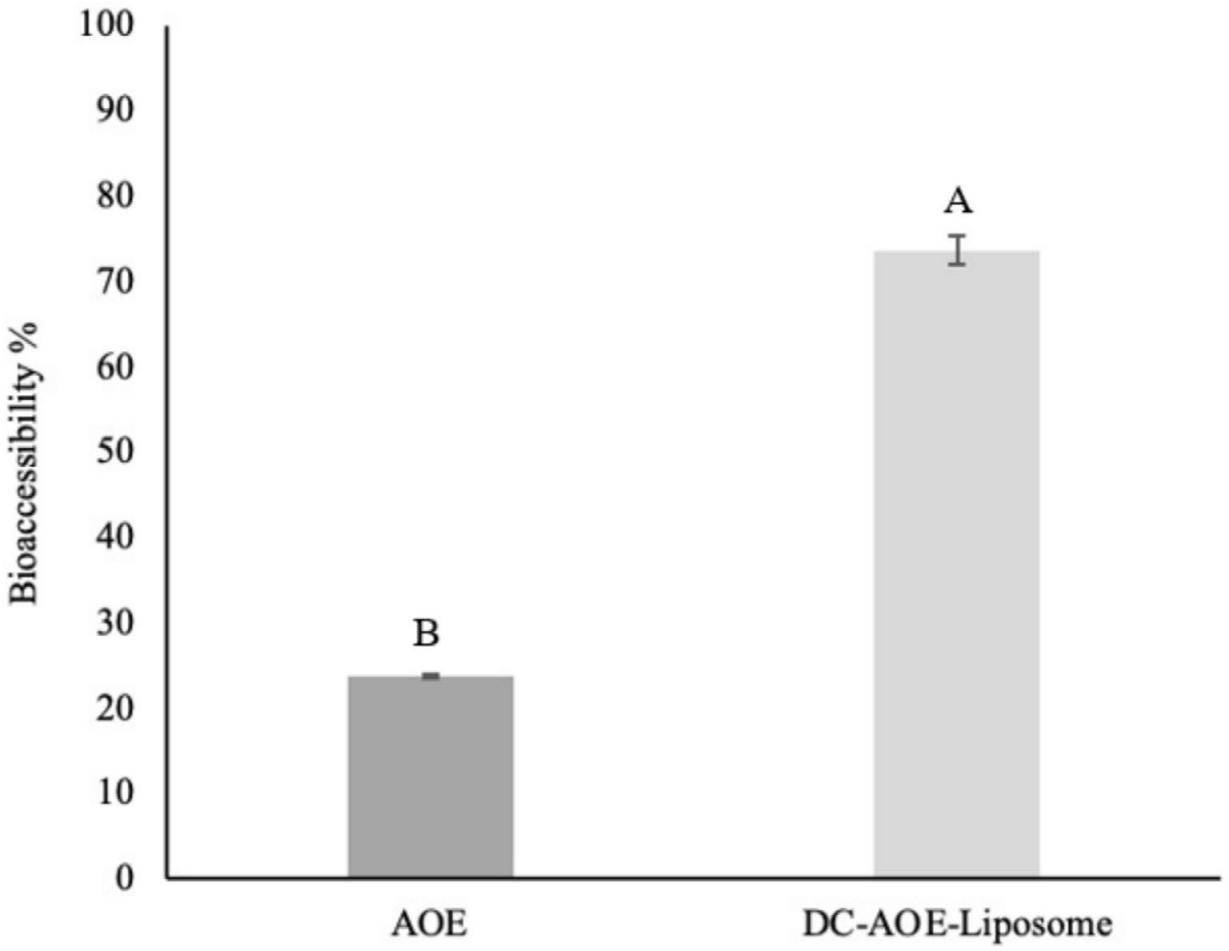
Bioaccessibility graph of AOE and DC‐AOE‐liposome. (A, B) Uppercase letter indicate significant differences between the bioaccessibility (%) of AOE and DC‐AOE‐liposome.

The bioaccessibility of galangin refers to the amount of galangin absorbed from the intestinal environment at the end of in vitro gastrointestinal digestion. The bioaccessibility (%) of galangin in AOE and DC‐AOE‐liposomes is given in Figure 5.

Bioaccessibility (%) of galangin after in vitro gastrointestinal digestion was determined as 23.87% ± 0.24% in AOE and 73.65% ± 1.70% in DC‐AOE‐liposomes. While 3.49 mg galangin was found in DC‐AOE‐liposomes and AOE at the beginning of in vitro gastrointestinal digestion, this amount was 2.57 mg with 73.65% bioaccessibility in DC‐AOE‐liposomes and 0.83 mg with 23.87% bioaccessibility in AOE after in vitro gastrointestinal digestion. Accordingly, there is a three‐fold difference between the amount of galangin theoretically absorbed from the intestines from AOE and DC‐AOE‐liposomes. Encapsulation of galangin into DC‐AOE‐liposomes resulted in a more than three fold increase in bioaccessibility. This indicates that galangin is made more accessible with the developed dual‐coated liposomal system. Systematically, bioaccessibility is the absorption of bioactive molecules from the intestines after oral, gastric, and intestinal digestion and subsequent entry into the bloodstream. After this stage, the molecules become bioavailable and can perform their biological activities in the body (Vlaicu et al. 2023). For this reason, encapsulation techniques such as liposomes are frequently applied to increase the bioaccessibility of bioactive components. It has been reported that especially lipophilic bioactive components are transported in the intestinal system by encapsulation, their permeability and absorption are increased, and thus their bioaccessibility is increased (He et al. 2025).

Similar to our study, Sengupta et al. (2023) encapsulated rutin, a flavonoid with low water solubility and bioavailability, into liposomes. They examined the bioaccessibility of rutin encapsulated in liposomes by in vitro gastrointestinal digestion study and determined that the bioaccessibility of free rutin was 9.8% ± 1.1%, while liposomal rutin was 19.7% ± 1.5%. Accordingly, a two ‐fold increase in bioaccessibility of rutin occurred with the encapsulation into liposomes. The point where this study differs from our study is that the lipid used in the liposome composition is a synthetic lipid. The resistance of synthetic lipids to in vitro gastrointestinal conditions is much higher than natural lipids. Therefore, the bioaccessibility level was increased more than three fold with the liposomes that we developed using natural lipids and applied surface modification. Rubaka et al. (2023) examined the bioaccessibility of *Carissa spinarum* extract in liposomes loaded with *Carissa spinarum* extract and in chitosan‐coated liposomes. The bioaccessibility of *Carissa spinarum* extract in the gastric medium was 39.65% ± 2.4%, 51.08% ± 3.2% in liposomes and 74.11% ± 1.3% in chitosan‐coated liposomes. In the intestinal medium, the bioaccessibility of *Carissa spinarum* extract enhanced from 31.4% ± 2.8% to 63.32% ± 5.3% in liposomes and 43.71% ± 3.3% in chitosan‐coated liposomes. The improvement in the bioaccessibility of *Carissa spinarum* extract has been linked to protection from oxidative degradation in the in vitro gastrointestinal medium in the presence of digestive enzymes. Digestive enzymes cause oxidation of the lipid components of liposomes and disruption of the structure. By means of surface modification with chitosan, the liposomal structure is absorbed from the intestines without being affected by digestive enzymes and pH, thus increasing the bioaccessibility of the components loaded in liposomes. According to the results obtained in this study, while the same amount of galangin was found in both AOE and DC‐AOE‐liposomes at the beginning, at the end of in vitro gastrointestinal digestion, DC‐AOE‐liposomes contained three times more galangin than AOE. This is because surface‐modified liposomes protect galangin from the digestive environment, as stated by Rubaka et al. (2023). Like our study, Cui et al. (2021) encapsulated chito‐oligosaccharides into uncoated liposomes, chitosan‐coated liposomes, and sodium alginate–chitosan‐coated liposomes by thin film hydration. When the release rate of chito‐oligosaccharides of the prepared liposomes in the in vitro simulated gastrointestinal system was examined, the release of chito‐oligosaccharides in gastric digestion showed a trend from fast to slow. They reported that the release rate of chito‐oligosaccharides in liposomes during gastric digestion was much higher than that of chitosan‐coated liposomes and sodium alginate–chitosan‐coated liposomes. They stated that this may be because sodium alginate may reduce pepsin activity, and therefore, sodium alginate–chitosan‐coated liposomes have higher stability in gastric mediums. During the in vitro simulated intestinal digestion process, liposomes without surface modification released more chito‐oligosaccharides than liposomes with surface modification. The minor release of chito‐oligosaccharides occurred in chitosan–alginate‐coated liposomes. They explained that the hydrolytic effect of trypsin on phospholipids in the intestinal medium can disrupt the structure of liposomes and cause the release of large amounts of chito‐oligosaccharides. Therefore, they stated that by modifying the surface of liposomes with chitosan and sodium alginate, liposomes can maintain their integrity by being protected from trypsin in the intestinal fluid, and the release of chito‐oligosaccharides can slow. Dong et al. (2023) modified fucoxanthin‐loaded liposomes with sodium alginate and chitosan and reported that the electrostatic interaction and weak hydrophobic force between chitosan and sodium alginate reduced the porosity and fluidity in the liposomal structure, preserved the liposome structure, slowed the release of fucoxanthin, protected it from the gastrointestinal digestion, and increased its bioaccessibility. The slowing of the release of bioactive components during gastrointestinal digestion also means that their bioaccessibility will increase.

## 4 Conclusion

*Alpinia officinarum* Hance extract has a substantial total phenolic‐flavonoid substance and antioxidant capacity. However, according to chromatographic analysis, the main component of the *Alpinia officinarum* extract was galangin, which has very poor solubility and intestinal absorption. In this case, it is expected that the bioaccessibility level of the extract will be low. Therefore, in this study, a layer‐by‐layer chitosan–sodium alginate coated liposomal carrier system resistant to the gastrointestinal digestive environment was developed to increase the bioaccessibility of the *Alpinia officinarum* Hance extract. The encapsulation efficiency of galangin in the developed DC‐AOE‐liposomes is relatively high, considered one of the most important factors for liposome production. In addition, the liposomes obtained have a spherical shape and a relatively average nanometric size, which is particularly important for stability and absorption from the body. The high negative zeta potential of liposomes was among the main objectives of this study. Liposomes having a zeta potential more negative than −30 mV indicates that they are physically stable, and the zeta potential of the obtained liposomes is suitable for this. However, the polydispersity index, which is another factor affecting stability, shows that liposomes have a very heterogeneous distribution, which is also confirmed by SEM and TEM images. This requires an additional homogenization step to ensure monodispersity in the liposome preparation process or an additional optimization step to determine the polymer concentration. In addition, in our previous study, a PDI value of 0.749 was obtained in chitosan–gum arabic coated liposomes. Changing the polymer type had positive effects on the zeta potential and size of liposomes, while it negatively affected the PDI value. This shows that the formulation can be improved by using different polymer/polymers combinations. In vitro release and gastrointestinal digestion study performed under simulated conditions to characterize the release behavior of liposomes show that the developed double layer liposomes provide control by slowing down the release of galangin and also protect it from different pH conditions. The bioaccessibility of galangin in double layer liposomes was increased three‐fold compared to galangin in the *Alpinia officinarum* Hance extract. However, the data provided by simulated conditions is the biggest limitation of the study and is open to confirmation by in vivo experiments. These results demonstrate that the developed DC‐AOE‐liposomes can be easily optimized/modified and used as carriers and are open to development for different active ingredients in various fields such as medicine and food due to the easy accessibility of the delivery system components.

## Author Contributions

**İlkyaz Patır:** conceptualization (equal), data curation (equal), formal analysis (equal), investigation (equal), methodology (equal), software (equal), validation (equal), visualization (equal), writing – original draft (equal), writing – review and editing (equal). **Büşra Karkar:** conceptualization (equal), data curation (equal), formal analysis (equal), software (equal), validation (equal), visualization (equal), writing – review and editing (equal). **Saliha Şahin:** conceptualization (equal), funding acquisition (equal), methodology (equal), project administration (equal), resources (equal), software (equal), supervision (equal), writing – review and editing (equal).

## Conflicts of Interest

The authors declare no conflicts of interest.

## Data Availability

The data that support the findings of this study are available on reasonable request from the corresponding author.
